# Link-N: The missing link towards intervertebral disc repair is species-specific

**DOI:** 10.1371/journal.pone.0187831

**Published:** 2017-11-08

**Authors:** Frances C. Bach, Lisanne T. Laagland, Michael P. Grant, Laura B. Creemers, Keita Ito, Björn P. Meij, Fackson Mwale, Marianna A. Tryfonidou

**Affiliations:** 1 Department of Clinical Sciences of Companion Animals, Faculty of Veterinary Medicine, Utrecht University, Utrecht, the Netherlands; 2 Department of Surgery, McGill University, Montreal, Canada; 3 Orthopedic Research Laboratory, Lady Davis Institute for Medical Research, SMBD-Jewish General Hospital, Montreal, Canada; 4 Department of Orthopedics, University Medical Center Utrecht, Utrecht, the Netherlands; 5 Orthopedic Biomechanics, Department of Biomedical Engineering, Eindhoven University of Technology, Eindhoven, the Netherlands; University of Hong Kong, HONG KONG

## Abstract

**Introduction:**

Degeneration of the intervertebral disc (IVD) is a frequent cause for back pain in humans and dogs. Link-N stabilizes proteoglycan aggregates in cartilaginous tissues and exerts growth factor-like effects. The human variant of Link-N facilitates IVD regeneration in several species *in vitro* by inducing Smad1 signaling, but it is not clear whether this is species specific. Dogs with IVD disease could possibly benefit from Link-N treatment, but Link-N has not been tested on canine IVD cells. If Link-N appears to be effective in canines, this would facilitate translation of Link-N into the clinic using the dog as an *in vivo* large animal model for human IVD degeneration.

**Materials and methods:**

This study’s objective was to determine the effect of the human and canine variant of Link-N and short (s) Link-N on canine chondrocyte-like cells (CLCs) and compare this to those on already studied species, *i*.*e*. human and bovine CLCs. Extracellular matrix (ECM) production was determined by measuring glycosaminoglycan (GAG) content and histological evaluation. Additionally, the micro-aggregates’ DNA content was measured. Phosphorylated (p) Smad1 and -2 levels were determined using ELISA.

**Results:**

Human (s)Link-N induced GAG deposition in human and bovine CLCs, as expected. In contrast, canine (s)Link-N did not affect ECM production in human CLCs, while it mainly induced collagen type I and II deposition in bovine CLCs. In canine CLCs, both canine and human (s)Link-N induced negligible GAG deposition. Surprisingly, human and canine (s)Link-N did not induce Smad signaling in human and bovine CLCs. Human and canine (s)Link-N only mildly increased pSmad1 and Smad2 levels in canine CLCs.

**Conclusions:**

Human and canine (s)Link-N exerted species-specific effects on CLCs from early degenerated IVDs. Both variants, however, lacked the potency as canine IVD regeneration agent. While these studies demonstrate the challenges of translational studies in large animal models, (s)Link-N still holds a regenerative potential for humans.

## Introduction

Low back pain affects up to 85% of the human population at some point during their lives, and this results in major socioeconomic consequences [1, 2]. Degeneration of the intervertebral disc (IVD) is frequently associated with low back pain [3]. The healthy IVD consists of a central, gelatinous nucleus pulposus (NP), fibrous annulus fibrosus, and cartilaginous endplates. During IVD maturation, the vacuolated notochordal cells (NCs) are gradually replaced by smaller chondrocyte-like cells (CLCs). When the IVD degenerates, the CLCs are not able to maintain healthy NP tissue anymore: CLCs become senescent, the glycosaminoglycan (GAG) content decreases and collagen type II is replaced by collagen type I, resulting in a more fibrous NP tissue with decreased swelling pressure. The avascular IVD exhibits inadequate matrix repair, and a vicious circle develops in which the IVD experiences increased vulnerability to damage by physiologic loading [4].

Current treatments for low back pain aim at relieving symptoms rather than restoring IVD function. Therefore, regenerative agents stimulating biological repair of the IVD (e.g. cell transplantation and growth factors) have gained interest [5, 6]. Several regenerative agents have been shown to successfully decrease cell apoptosis, stimulate chondrogenic extracellular matrix (ECM) production, and/or enhance mesenchymal stromal cell (MSC) differentiation to an NP-like phenotype, but disadvantages are high costs and potential side-effects [7, 8]. Several of these regenerative strategies, e.g. mesenchymal precursor cell (NCT01290367)/disc chondrocyte (NCT01640457) transplantation and growth factor application (NCT00813813) have entered the clinical trial phase, but no effective regenerative therapy for IVD degeneration is clinically available yet. Therefore, there is need for identifying new therapeutic agents that can induce IVD regeneration.

A promising alternative agent that can be produced synthetically, and is therefore relatively cheap, is Link-N peptide. Link-N (DHLSDNYTLDHDRAIH) is the N-terminal peptide of the link protein that stabilizes proteoglycan aggregates in the IVD and cartilage. It is generated *in vivo* by proteolytic degradation during tissue turnover [8–11]. The human variant of Link-N peptide has been demonstrated to stimulate GAG and/or collagen production *in vitro* in rabbit [12], human [7, 11, 13], and bovine [7, 9, 13] IVD cells, and degenerated bovine IVDs [14]. Furthermore, it exerted regenerative effects on experimentally induced degenerated rabbit IVDs [8], but has not yet been tested *in vivo* on large animal models. It is known that Link-N exerts its growth factor-like effects on rabbit NCs via the bone morphogenetic protein receptor type II (BMPRII), inducing a complex, positive Smad1/5/8 feedback loop [12], but this has not been investigated in other species yet. Previous work indicated that Link-N is cleaved by AF cells, creating a peptide consisting of only the first eight amino acids of full-length Link-N (DHLSDNYT) [13]. This small peptide, named short Link-N (sLink-N), induced GAG synthesis in both human and bovine IVD cells to a similar extent as full-length Link-N [13] and repaired bovine IVDs in which degeneration was induced in a whole organ culture model [15].

Altogether, Link-N and sLink-N may be promising candidates for the treatment of patients with IVD disease. Since dogs also experience back pain related to IVD degeneration, the dog is a particularly clinically relevant animal model [16]. Given that both species show similar pathophysiologic mechanisms of IVD degeneration, treatment strategies can be approached by the “One Medicine” concept: advances in the biomedical field of IVD regeneration hold a future, also for the veterinary patient. However, thus far, the effect of (short) Link-N on canine IVDs has not been delineated yet. Therefore, the objective of this study was to determine the effect of (short) Link-N on canine CLCs and compare the effects to those on already studied species, i.e. human and bovine CLCs. Our hypothesis was that human, and especially canine (short) Link-N would induce ECM production in canine CLCs. If human/canine (short) Link-N indeed appears to be effective in canine CLCs, this would facilitate the translation of (short) Link-N into the clinic using the dog as an *in vivo* large animal model for human IVD degeneration. Additionally, it would pave the way to an effective and affordable regenerative treatment for both human and canine patients with IVD disease.

## Materials and methods

### IVD collection and CLC isolation

CLCs from human (Thompson score III), bovine (Thompson score II), and chondrodystrophic (CD) and non-chondrodystrophic (NCD) canine (Thompson score II-III) donors were collected from early degenerated IVDs as described previously [17]. Briefly, NP tissue was enzymatically digested with 0.15% pronase (11459643001, Roche Diagnostics) for 45 minutes and 0.15% collagenase type II (4176, Worthington) for 15 hours at 37°C. After digestion, 100% CLCs, and no NCs were present in all species. Human IVDs were obtained during standard post mortem diagnostics. The L2-L5 part of the spine was collected (≤48 hours after death), as approved by the scientific committee of the Pathology department of the University Medical Centre Utrecht (UMCU). Anonymous use of redundant tissue for research purposes is a standard treatment agreement with UMCU patients (Local Medical Ethical Committee number 12–364). The material was used in line with the code ‘Proper Secondary Use of Human Tissue’, installed by the Federation of Biomedical Scientific Societies. Bovine tails were collected from the slaughterhouse (Nederlandse Voedsel- en Warenautoriteit, Utrecht, the Netherlands, permit number 457642.09) and IVDs from complete canine spines were collected from dogs euthanized in unrelated research studies [18], approved by the Utrecht University Animal Ethics Committee (experimental numbers: 2012.III.07.065, 2013.III.02.017, and 2013.II.12.126).

### Cell culture

Since culturing IVD cells in a 3D environment maintains their disc phenotype better than 2D culture [19, 20] and canine cells do not thrive in alginate beads [21], CLC micro-aggregates were used to determine the effect of (s)Link-N. Previous *in vitro* studies demonstrating regenerative effects of (s)Link-N were performed in human or bovine monolayer [11, 22] or alginate bead [7, 13] cultures. To allow inter-species comparison in this study, canine, human, and bovine CLC micro-aggregate cultures were used.

One million CLCs from three human (47, 63, and 67 years of age), six bovine (2 years of age), six CD canine (2–6 years of age, Beagles) and six NCD canine (4–11 years of age, 3 German shepherds, 1 Cocker Spaniël, 1 Greyhound, and 1 Irish Setter) donors were expanded as described previously [17] in expansion medium containing hgDMEM+Glutamax (31966, Invitrogen) with 10% FBS (16000–044, Life Technologies), 1% penicillin/streptomycin (P/S, P11-010, PAA Laboratories), 0.1 mM Ascorbic acid 2-phosphate (Asap, A8960, Sigma-Aldrich), 10^−9^ M dexamethasone (AD1756, Sigma-Aldrich) and 1 ng/mL bFGF (PHP105, AbD Serotec). After expansion for two (bovine, canine) or three (human) passages, 35,000 CLCs were plated per well in low-adherence cell-repellent surface 96-well plates (650970, CELLSTAR^®^ Greiner Bio-one) in 50 μL basal culture medium: hgDMEM+Glutamax, 1% P/S, 1% ITS+ premix (354352, Corning Life Sciences), 0.04 mg/mL L-proline (P5607, Sigma-Aldrich), 0.1 mM Asap, and 1.25 mg/mL Bovine Serum Albumin (A9418, Sigma-Aldrich). The 96-well plates were centrifuged at 50*g* for 5 minutes to induce micro-aggregate formation. The next day, basal culture medium was replaced with basal culture medium (negative controls) or supplemented with (a) 10 ng/mL human recombinant TGF-β_1_ (240-B, R&D Systems), (b) 1 μg/mL or 10 ng/mL human Link-N (DHLSDNYT-LDHDRAIH, CanPeptide), (c) 0.5 μg/mL or 5 ng/mL human sLink-N (DHLSDNYT, CanPeptide), (d) 1 μg/mL or 10 ng/mL canine Link-N (DHHSDNYT-LNYDVIH, CanPeptide), or (e) 0.5 μg/mL or 5 ng/mL canine sLink-N (DHHSDNYT, CanPeptide). Culture medium was replaced twice a week and (s)Link-N was supplemented every medium change. (s)Link-N concentrations were chosen based upon previous work [9, 13, 14] and on a monolayer pilot study with CD canine CLCs (S1 Fig). Since canine CLCs do not produce a considerable amount of GAGs if no growth factor is supplied, TGF-β_1_ was used to show that the canine CLCs were able to produce GAGs if a proper stimulus was provided. Initially, the micro-aggregates were cultured for 28 days at 21% O_2_, 5% CO_2_, 37°C to determine the effects of human (s)Link-N. Follow-up culture experiments (i.e. using canine (s)Link-N) where performed at 5% O_2_, 5% CO_2_, 37°C, to improve the chondrogenic response of the CLCs [23, 24].

### Read out parameters for the biologic effect of (s)Link-N at the matrix level

At day 28, micro-aggregates were collected for determining the GAG and DNA content (in duplicates). Sample preparation was performed as described previously [17]. Papain digestion solution (pH 6, 200 mM H_2_NaPO_4_*2 H2O (21254, Boom B.V.), 10 mM EDTA (100944, Merck Millipore), 10 mM cysteine HCl (C7880, Sigma-Aldrich), and 10 mM papain (P3125, Sigma-Aldrich) was added to each micro-aggregate, followed by overnight incubation at 60°C. The micro-aggregates’ GAG content and release in the culture medium (cumulative GAG release over the 28 day period) was measured using a dimethyl methylene blue (DMMB) assay [25]. Immediately after DMMB (341088, Sigma-Aldrich) was added, the absorbance (540/595 nm) was measured using a microplate reader. The GAG content was calculated using a chondroitin sulphate (C4384, Sigma-Aldrich) standard line with polynomic properties. DNA content was measured using the Qubit^®^ dsDNA High Sensitivity Assay Kit (Q32851, Invitrogen) according to the manufacturer’s instructions.

Also at day 28, micro-aggregates were fixed in 4% neutral buffered formaldehyde for 24 hours and embedded in alginate and paraffin (in duplicates). Five μm sections were mounted and Safranin O/Fast Green staining, and collagen type I and II immunohistochemistry were performed as described previously [17]. The primary antibodies for collagen type I (human and bovine: 0.1 μg/mL, canine: 0.07 μg/mL; ab6308, Abcam) and II (human and bovine: 0.4 μg/mL, canine: 0.02 μg/mL; II-II6B3, DSHB) were applied with adjusted concentrations per species. In isotype controls, normal mouse IgG_1_ (3877, Santa Cruz) employed at the same concentration as the primary antibody showed no staining.

### BMP receptor expression and activation of Smad signaling by (s)Link-N

At day 7, *BMPRIa*, *BMPRIb*, and *BMPRII* gene expression was determined in CD canine CLC micro-aggregates treated with control culture medium supplemented with/without 10 ng/mL TGF-β_1_, 1 μg/mL or 10 ng/mL canine Link-N, or 0.5 μg/mL or 5 ng/mL canine sLink-N. The micro-aggregates were frozen in liquid nitrogen and crushed using pestles (P9951-901, Argos Technologies). RNA was extracted with the RNeasy^®^ Micro kit (74004, Qiagen) according to the manufacturer’s instructions. A DNase (RNAse-Free DNase Set, 79254, Qiagen) step was included to ensure DNA removal. The quality of the isolated RNA was assessed with an Agilent 2100 Bioanalyzer and RNA Nanochip kit (5067–1511, Agilent Technologies). cDNA was synthesized using the iScript^™^ cDNA Synthesis Kit (170–8891, Bio-Rad) according to the manufacturer’s instructions. Primer sequences were designed using PerlPrimer (http://perlprimer.sourceforge.net). M-fold was used to check for secondary structure formation [26]. Primer uniqueness and specificity was determined using BLAST [27]. Annealing temperatures were established by performing a temperature gradient PCR on a 16-fold dilution series. The four most stably expressed reference genes were chosen to normalize gene expression of the target genes (Table 1).

**Table 1:** Primers used for quantitative PCR.

Genes	Forward sequence 5’ → 3’	Reverse sequence 5’ → 3’	Amplicon size	Annealing temp (°C)
**Reference genes**				
*GAPDH*	TGTCCCCACCCCCAATGTATC	CTCCGATGCCTGCTTCACTACCTT	100	58
*HPRT*	AGCTTGCTGGTGAAAAGGAC	TTATAGTCAAGGGCATATCC	104	58
*RPS19*	CCTTCCTCAAAAAGTCTGGG	GTTCTCATCGTAGGGAGCAAG	95	61
*SDHA*	GCCTTGGATCTCTTGATGGA	TTCTTGGCTCTTATGCGATG	92	56.5
**Target genes**				
*BMPRIa*	TTTGGGAAATGGCTCGTC	CGTATGATGGATCGTTGGG		60
*BMPRIb*	CCCTATCATGACCTAGTGCC	TGCCTCAGACACTCATCAC		63
*BMPRII*	GTCTTCACAGTATGAACATGATGG	AACACTTTCACAGCAACTGG	150	64

*GAPDH*: glyceraldehyde 3-phosphate dehydrogenase, *HPRT*: hypoxanthine-guanine phosphoribosyltransferase, *RPS19*: ribosomal protein S19, *SDHA*: succinate dehydrogenase subunit A.

RT-qPCR was performed using the iQTTM SYBR Green Supermix Kit (Bio-Rad) and the CFX384 Touch^™^ Real-Time PCR Detection System (Bio-Rad) (40 cycles; denaturation 95°C, annealing temp (Table 1), extension 65°C). For determination of relative quantitative gene expression, the Normfirst (E^ΔΔCq^) method was used. For each target gene, the Cq-value of the test sample and the calibrator sample was normalized to the mean Cq-value of the reference genes: ΔCq = Cqmean ref−Cq_target_. Cq-values of the negative control micro-aggregates were used as calibrator. Secondly, the E^ΔCq^-value for the test and calibrator sample was calculated. In this formula, E indicates the amplification efficiency of the target/reference gene. E^ΔΔCq^ was calculated by normalizing the E^ΔCq^-value of the test sample to the one of the calibrator: E^ΔΔCq^ = E^ΔCq^ test − E^ΔCq^ calibrator. For each target gene, the mean n-fold changes and standard deviations in gene expression were calculated.

ELISAs for phosphorylated (p) Smad1 (SER463/465, PEL-SMAD1-S463, RayBiotech) and pSmad2 (S245/250/255, PEL-SMAD2-S245, RayBiotech) were used to determine activation of the BMP and TGF-β Smad signaling pathway. For this purpose, 200,000 CLCs from five human (44, 47, 47, 63, and 67 years of age), five bovine (2 years of age), and five canine (Beagles, 2–6 years of age) donors were plated per well (12-wells plate, 665180, Greiner CELLSTAR^®^) in expansion medium, which was replaced after 2 days with basal culture medium alone or supplemented with 10 ng/mL TGF-β_1_, 250 ng/mL BMP2 (TETEC AG), 1 μg/mL human Link-N, 1 μg/mL canine Link-N, 0.5 μg/mL human sLink-N or 0.5 μg/mL canine sLink-N. TGF-β_1_ and BMP2 served here as positive controls. After 24 hours (time point based on Wang *et al*., 2013 [12]), cells were homogenized in cell lysis buffer containing 0.6 mM phenylmethylsulphonyl fluoride, 17 μg/mL aprotinin and 1 mM sodium orthovanadate (Sigma-Aldrich). The data were corrected for the samples protein concentration, measured using the Qubit^®^ Protein Assay Kit (Q32851, Invitrogen).

### (Short) Link-N peptide structure prediction and docking

The human, canine and bovine models of (s)Link-N were generated using the PEP-FOLD3 server [28]. The first eight residues of human and canine/bovine Link protein were inputted and the best predicted model was used for docking. Human, canine, and bovine (s)Link-N molecular structures were viewed and aligned using PyMOL (The PyMOL Molecular Graphics System, Version 1.8, Schrӧdinger, Germany). Docking of (s)Link-N to BMPRII was performed using the CABS-dock server (www.biocomp.chem.uw.edu.pl/CABSdock/) and the crystal structure of the extracellular domain of BMPRII (PDB ID: 2HLR) [29] downloaded from the Protein Data Bank (www.rcsb.org). The original crystal structure of the BMPRII extracellular domain was from sheep, since there are no entries for the crystal structures of human, bovine, or canine BMPRII extracellular domains. The best-fit model with the lowest root-mean-square deviation (RMSD) was used for imaging with PyMOL.

### Statistical analysis

Statistical analyses were performed using IBM SPSS statistics 22. Data were examined for normal distribution with the Shapiro Wilks test. General linear regression models based on ANOVAs were used for normally distributed data, whereas Kruskal Wallis and Mann-Whitney U tests were used for non-normally distributed data. Benjamini & Hochberg False Discovery Rate *post-hoc* tests were performed to correct for multiple comparisons. *p*-values < 0.05 were considered significant.

## Results

### Human (s)Link-N induced GAG and collagen type I deposition by human CLCs

The first objective of this study was to determine the effects of human (s)Link-N on canine CLCs and to compare those to already studied species. Therefore, the effects of human (s)Link-N were initially determined on human and bovine CLC micro-aggregates.

**Fig 1:**
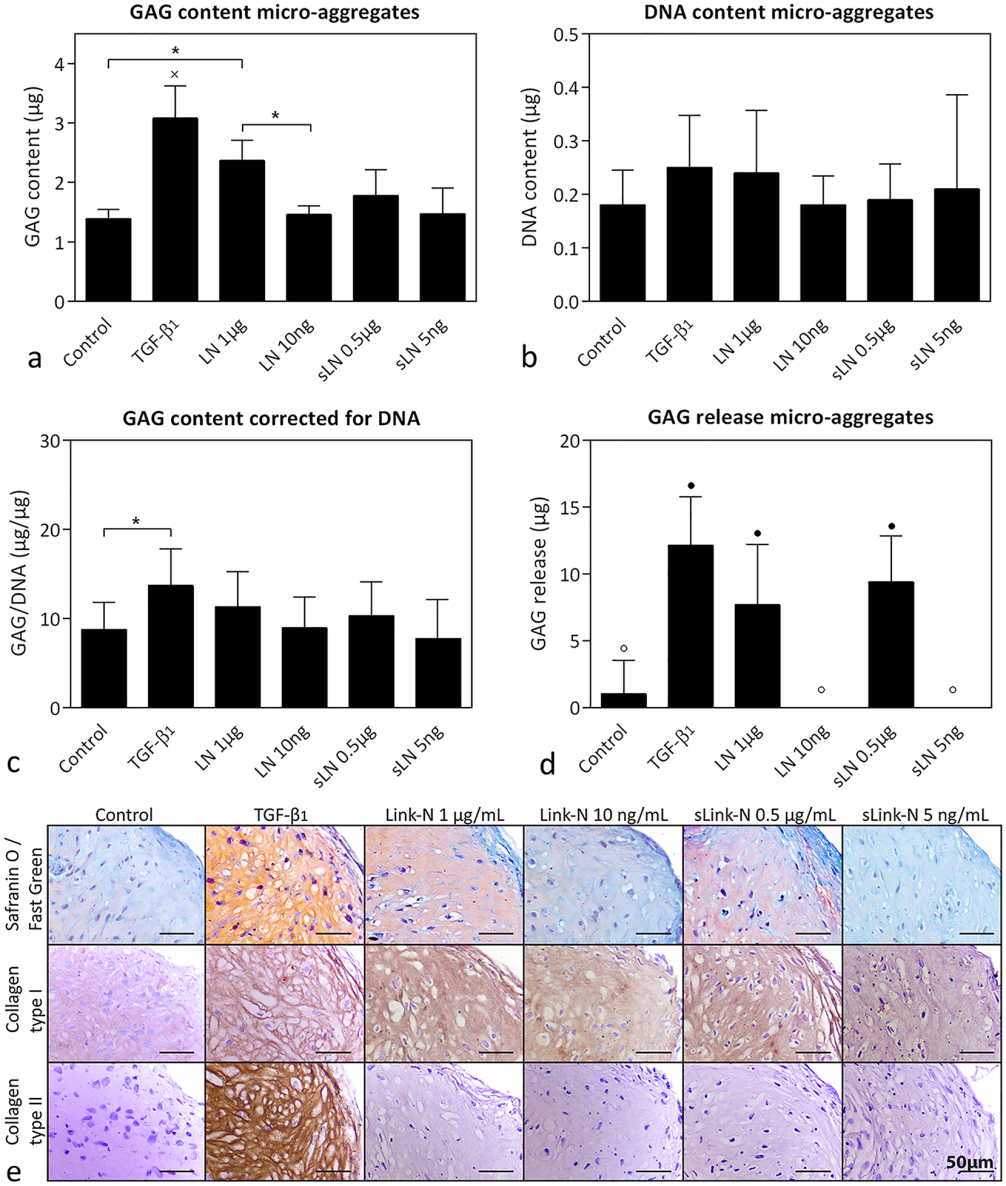
Effect of human (short) Link-N on human chondrocyte-like cells (CLCs). GAG and DNA content (mean + SD) and histological evaluation of human CLC micro-aggregates treated with basal culture medium (control), supplemented with 10 ng/mL TGF-β_1_ (positive control), 1 μg/mL or 10 ng/mL human Link-N (LN), or 0.5 μg/mL or 5 ng /mL human short Link-N (sLN) for 28 days in normoxia (21% O_2_). (**a**) GAG content, (**b**) DNA content, (**c**) GAG content (incorporation in the micro-aggregate) corrected for DNA content, (**d**) total amount of GAGs released in the culture medium, (**e**) representative histological images of the Safranin O/Fast Green staining and collagen type I and II immunohistochemistry. *: *p* < 0.05; ● and ○: significantly different (*p* < 0.05) from all other conditions except for the bars with the same symbol; x: significantly different (*p* < 0.05) from all other conditions. *n* = 3 (*in duplicates*).

TGF-β_1_ treatment resulted in the highest GAG content of the human CLC micro-aggregates after 28 days (*p*<0.05; Fig 1a). Treatment with 1 μg/mL human Link-N also resulted in a significantly increased GAG content of the micro-aggregates compared with control and 10 ng/mL human Link-N treatment (*p*<0.05), indicating a concentration-dependent effect. This was confirmed by Safranin O/Fast Green staining (Fig 1e). No treatment affected the DNA content of the micro-aggregates (Fig 1b). Only TGF-β_1_ upregulated the GAG/DNA content (indication of GAG incorporation in the micro-aggregate per cell) compared with controls (*p*<0.05; Fig 1c). GAG release into the culture medium was significantly increased by TGF-β_1,_ 1 μg/mL human Link-N and 0.5 μg/mL human sLink-N treatment compared with control, 10 ng/mL human Link-N, and 5 ng/mL human sLink-N treatment (*p*<0.05; Fig 1d). GAG incorporation (GAG content micro-aggregate divided by total GAG production micro-aggregate) was, however, not significantly different between control, TGF-β_1_, 1 μg/mL human Link-N and 0.5 μg/mL human sLink-N treatment (S2A Fig). Both TGF-β_1_ and human (s)Link-N induced collagen type I deposition, whereas only TGF-β_1_ increased collagen type II deposition compared with controls (Fig 1e).

### Human (s)Link-N induced GAG and collagen type I deposition by bovine CLCs

**Fig 2:**
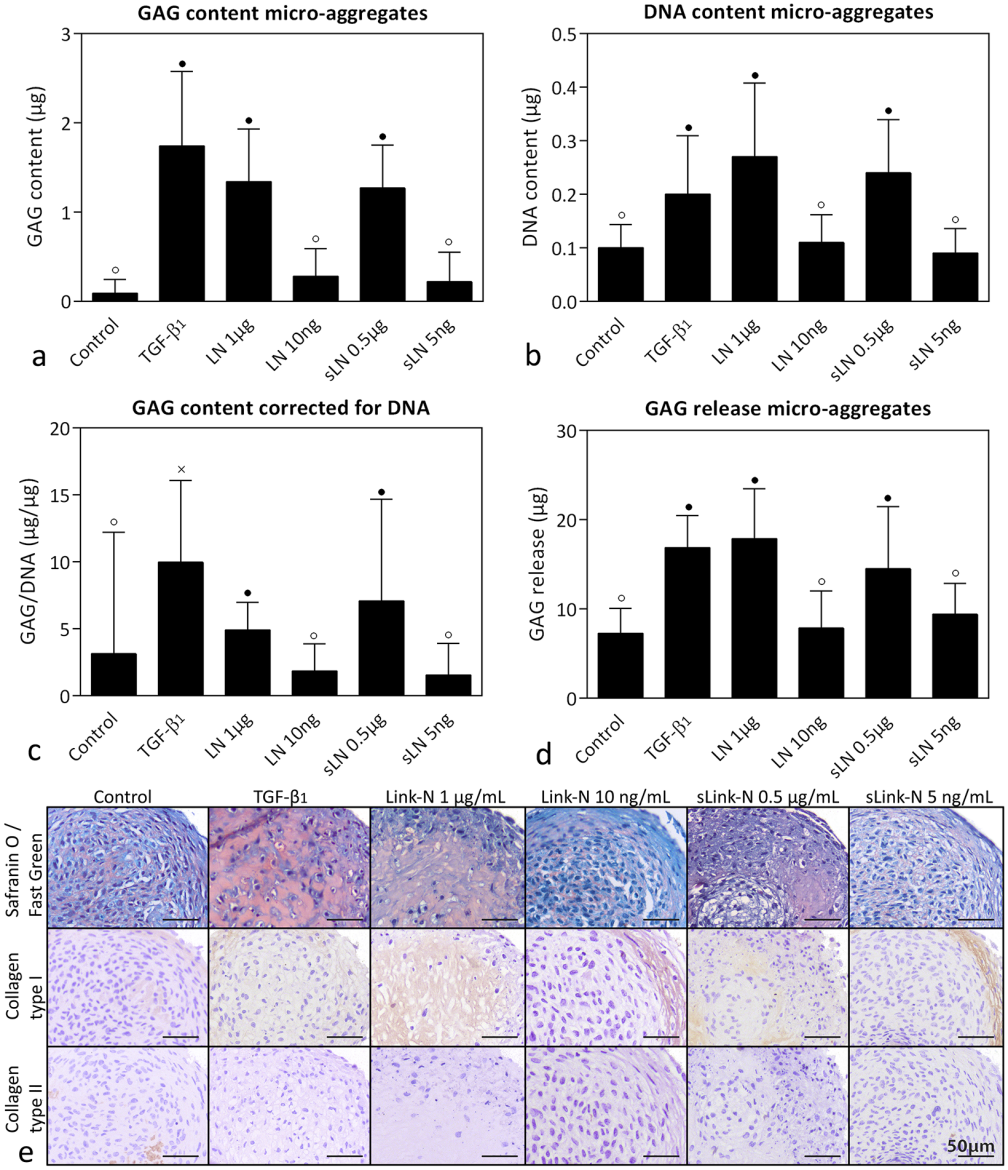
Effect of human (short) Link-N on bovine chondrocyte-like cells (CLCs). GAG and DNA content (mean + SD) and histological evaluation of bovine CLC micro-aggregates treated with basal culture medium (control), supplemented with 10 ng/mL TGF-β_1_, 1 μg/mL or 10 ng/mL human Link-N (LN), or 0.5 μg/mL or 5 ng/mL human short Link-N (sLN) for 28 days in normoxia (21% O_2_). (**a**) GAG content, (**b**) DNA content, (**c**) GAG content (incorporation in the micro-aggregate) corrected for DNA content, (**d**) total amount of GAGs released in the culture medium, (**e**) representative histological images of the Safranin O/Fast Green staining and collagen type I and II immunohistochemistry. ● and ○: significantly different (*p* < 0.05) from all other conditions except for the bars with the same symbol; x: significantly different (*p* < 0.05) from all other conditions. *n* = 6 (*in duplicates*).

Treatment with TGF-β_1,_ 1 μg/mL human Link-N, and 0.5 μg/mL human sLink-N resulted in a significantly higher GAG, DNA, and GAG/DNA content, GAG incorporation percentage and GAG release of the bovine CLC micro-aggregates compared with control, 10 ng/mL human Link-N, and 5 ng/mL human sLink-N treatment (*p*<0.05; Fig 2a–2d and S2C Fig), indicating a concentration-dependent effect. This was confirmed by Safranin O/Fast Green staining (Fig 2e). A rim of collagen type I was detected around the micro-aggregates treated with 10 ng/mL human Link-N and 5 ng/mL human sLink-N, while 1 μg/mL human Link-N and 0.5 μg/mL human sLink-N treatment induced collagen type I deposition in the center of the micro-aggregates (Fig 2e). Deposition of collagen type II was not observed with TGF-β_1_ or human Link-N treatment (Fig 2e).

### Human (s)Link-N induced negligible GAG deposition by CD canine CLCs

**Fig 3:**
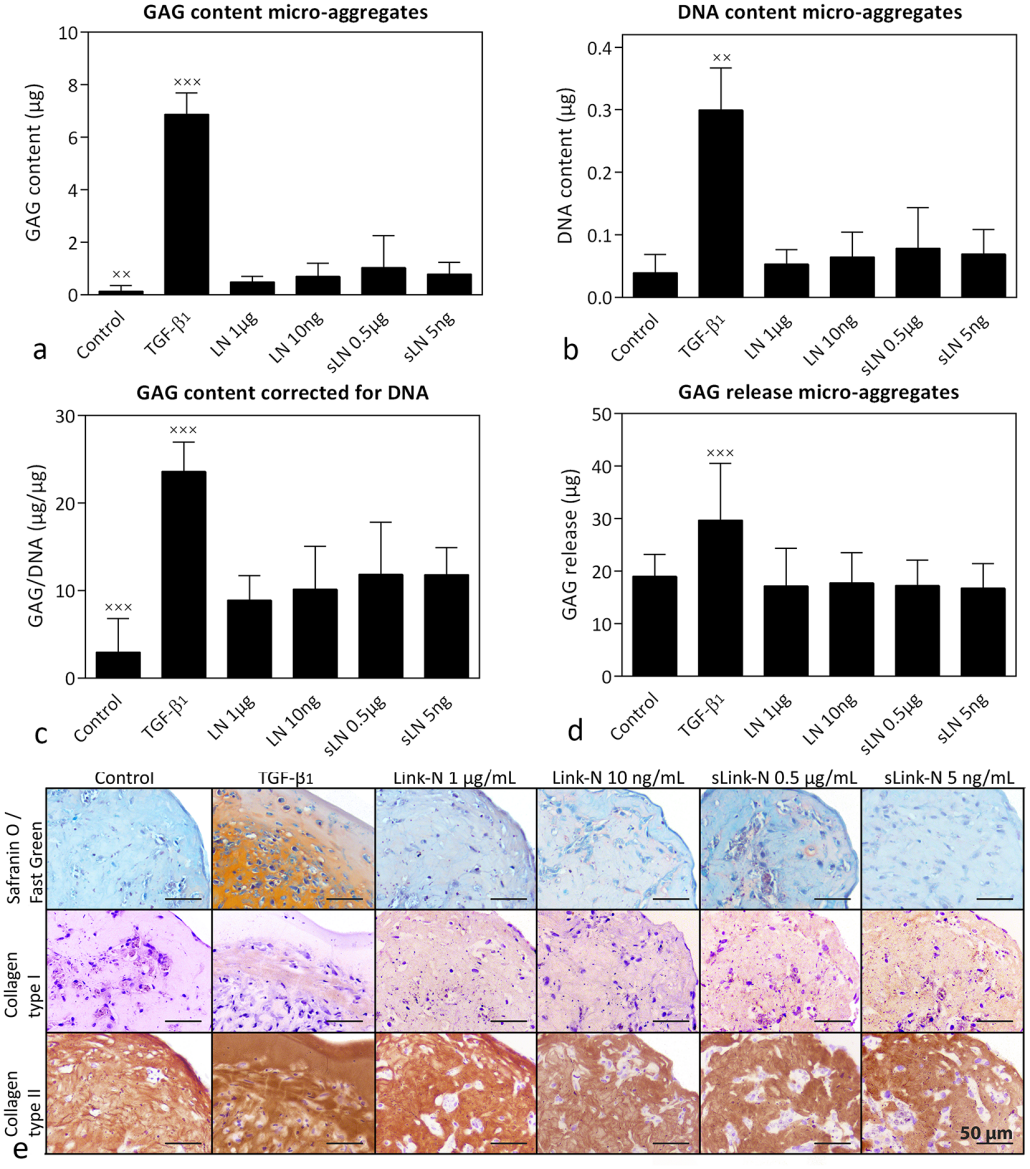
Effect of human (short) Link-N on CD canine chondrocyte-like cells (CLCs). GAG and DNA content (mean + SD) and histological evaluation of CD canine CLC micro-aggregates treated with basal culture medium (control), supplemented with 10 ng/mL TGF-β_1_, 1 μg/mL or 10 ng/mL human Link-N (LN), or 0.5 μg/mL or 5 ng/mL human short Link-N (sLN) for 28 days in normoxia (21% O_2_). (**a**) GAG content, (**b**) DNA content, (**c**) GAG content (incorporation in the micro-aggregate) corrected for DNA content, (**d**) total amount of GAGs released in the culture medium, (**e**) representative histological images of the Safranin O/Fast Green staining and collagen type I and II immunohistochemistry. xx,xxx: significantly different (*p* < 0.01 and *p* < 0.001 respectively) from all other conditions. *n* = 6 (*in duplicates*).

Although far less potent as TGF-β_1_, human (s)Link-N treatment at all concentrations significantly increased the GAG content of the CD canine CLC micro-aggregates compared with controls, both corrected (*p*<0.001) and not corrected (*p*<0.01) for the DNA content (Fig 3a and 3c). Additionally, human (s)Link-N at all concentrations also significantly increased GAG incorporation compared with controls (*p*<0.05; S2E Fig). TGF-β_1_ induced the highest DNA content, GAG release and GAG incorporation compared with all the other conditions (*p*<0.01), whereas human (s)Link-N did not affect the DNA content and GAG release compared with controls (Fig 3b and 3d).

In line with the biochemical data, Safranin O/Fast Green staining demonstrated limited GAG deposition in the human (s)Link-N-treated micro-aggregates (Fig 3e). Addition of TGF-β_1_ resulted in most GAG deposition and a cell-depleted rim around the micro-aggregates. Collagen type I deposition was slightly induced by TGF-β_1_ and human (s)Link-N, while no differences in collagen type II deposition were detected between conditions (Fig 3e).

Given that a lower O_2_ tension has been shown to facilitate ECM deposition [23], the effect of human (s)Link-N on CD canine CLCs was also studied under hypoxia (S3 Fig). It appeared that the effects of human (s)Link-N were not affected by the O_2_ tension: in both conditions, human (s)Link-N exerted a limited anabolic effect compared with controls. Nevertheless, since the IVD is an avascular structure, hypoxia better mimics the *in vivo* situation and has been shown to better preserve the regenerative potential of CLCs [23, 24], follow-up experiments (e.g. using canine (s)Link-N) were continued in hypoxia.

### Species differences in amino acid sequence of (short) Link-N

**Fig 4:**
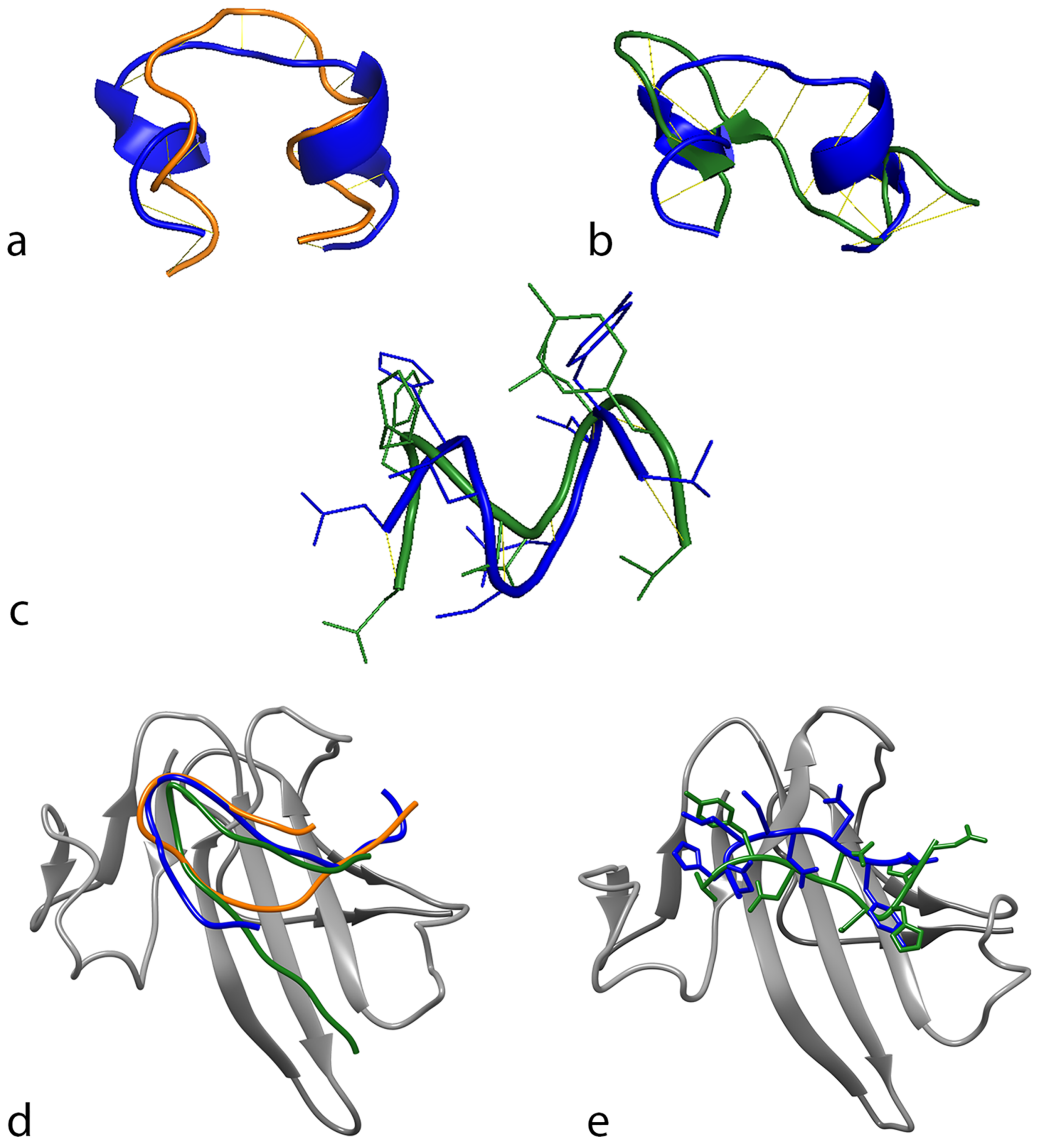
Alignment and docking of human, bovine, and canine (short) Link-N variants. Schematic of the predicted molecular model and alignment of human (blue) and bovine (orange) Link-N (**a**) and human (blue) and canine (green) Link-N (**b**). (**c**) Schematic of the predicted molecular model and alignment of human (blue) and canine/bovine (green) short Link-N. (**d**) Docking of human (blue), bovine (orange) and canine (green) Link-N to the extracellular domain of BMPRII. (**e**) Docking of human (blue) and canine/bovine (green) short Link-N to the extracellular domain of BMPRII. Models represent best-fit predictions for their interaction.

A possible explanation for the limited response of the canine CLCs to human (s)Link-N could be differences in amino acid sequence between human and canine (s)Link-N. Indeed, alignment of human, bovine and canine Link-N revealed that the amino acid sequence of canine Link-N differed by five residues and bovine Link-N by three residues when compared to human Link-N (Table 2). Furthermore, the predicted molecular structures of bovine Link-N and canine Link-N also revealed differences when aligned with human Link-N (Fig 4a and 4b). The amino acid sequences of canine and bovine sLink-N are similar and differ by only one amino-acid from human sLink-N (Table 2). A schematic of the predicted molecular structures of human and canine/bovine sLink-N show that these amino acid substitutions influence the conformation of the peptide (Fig 4c). Interestingly, when we prepared docking simulations of all three Link-N species to the extracellular domain of BMPRII, bovine, canine, and human Link-N docked differently (Fig 4d). Docking differences were also observed for human and canine/bovine sLink-N variants (Fig 4e).

**Table 2:** Alignment of human, bovine, and canine (short) Link-N peptides.

Residue	1	2	3	4	5	6	7	8	-	9	10	11	12	13	14	15	16
**Human**	D	H	L	S	D	N	Y	T	-	L	D	H	D	R	A	I	H
**Bovine**	D	H	**H**	S	D	N	Y	T	-	**V**	D	H	D	R	**V**	I	H
**Canine**	D	H	**H**	S	D	N	Y	T	-	**V**	**N**	**Y**	D	R	**V**	I	H

Short Link-N represents residues 1–8. Bovine and canine short Link-N share similarity.

### Canine (s)Link-N did not affect ECM production by human CLCs

**Fig 5:**
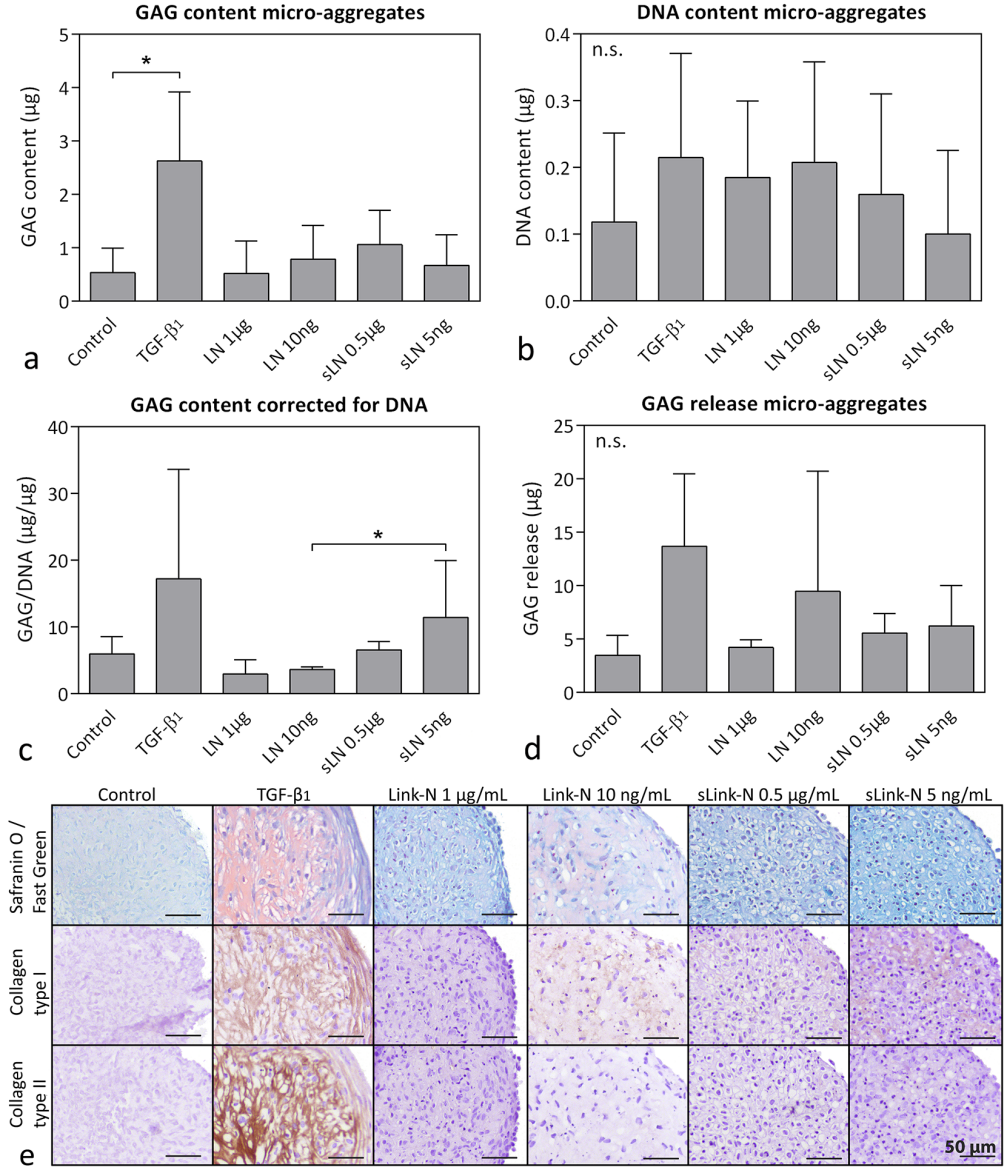
Effect of canine (short) Link-N on human chondrocyte-like cells (CLCs). GAG and DNA content (mean + SD) and histological evaluation of human CLC micro-aggregates treated with basal culture medium (control), supplemented with 10 ng/mL TGF-β_1_, 1 μg/mL or 10 ng/mL canine Link-N (LN), or 0.5 μg/mL or 5 ng/mL canine short Link-N (sLN) for 28 days in hypoxia (5% O_2_). (**a**) GAG content, (**b**) DNA content, (**c**) GAG content (incorporation in the micro-aggregate) corrected for DNA content, (**d**) total amount of GAGs released in the culture medium, (**e**) representative histological images of the Safranin O/Fast Green staining and collagen type I and II immunohistochemistry. *: *p* < 0.05. *n* = 3 (*in duplicates*).

Since we detected species differences in amino acid sequence and receptor docking of human and canine (s)Link-N which could possibly explain the limited response of canine CLCs to human (s)Link-N, the effect of canine (s)Link-N was determined on human, bovine and canine CLCs. As under normoxic culture conditions, TGF-β_1_ induced the GAG content of human CLC micro-aggregates cultured under hypoxic conditions (*p*<0.05; Fig 5a), which was confirmed by Safranin O/Fast Green staining (Fig 5e). In contrast, canine (s)Link-N treatment did not augment GAG deposition compared with controls (Fig 5a). No treatment affected the DNA content of the micro-aggregates (Fig 5b). The GAG/DNA content of the micro-aggregates treated with 5 ng/mL canine sLink-N was significantly higher than those treated with 10 ng/mL canine Link-N (*p*<0.05), but no treatment affected the GAG/DNA content compared with controls (Fig 5c). There were no significant effects of canine (s)Link-N or TGF-β_1_ on GAG release compared with controls (Fig 5d). Also, GAG incorporation percentages were not significantly different between the conditions (S2B Fig). The deposition of collagen type I was prominently increased by TGF-β_1_ and slightly increased by 10 ng/mL canine Link-N and 5 ng/mL canine sLink-N compared with controls (Fig 5e). TGF-β_1_ increased collagen type II deposition, whereas this was not affected by canine (s)Link-N (Fig 5e).

### Canine (s)Link-N mainly induced collagen type I and II deposition by bovine CLCs

**Fig 6:**
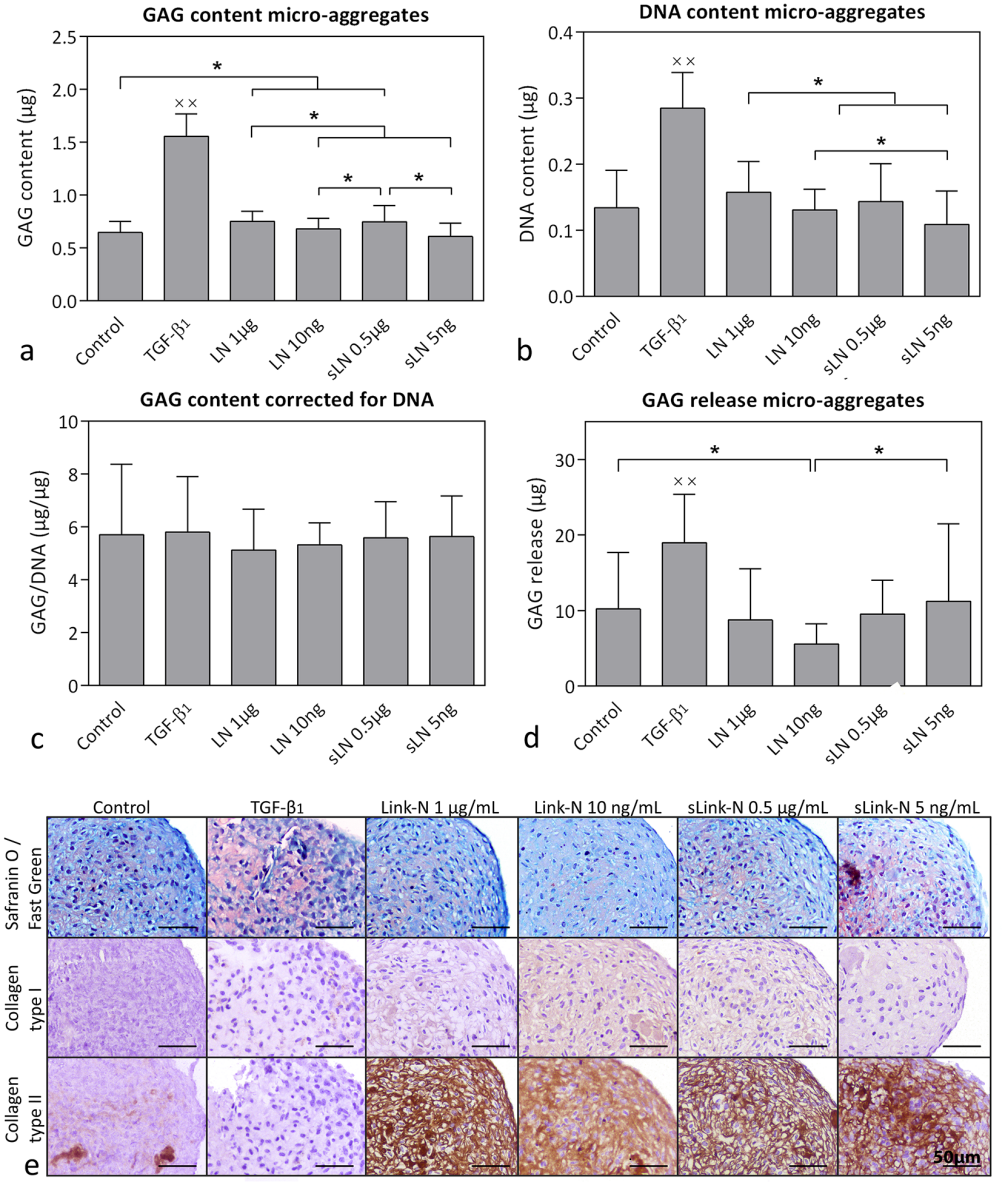
Effect of canine (short) Link-N on bovine chondrocyte-like cells (CLCs). GAG and DNA content (mean + SD) and histological evaluation of bovine CLC micro-aggregates treated with basal culture medium (control), supplemented with 10 ng/mL TGF-β_1_, 1 μg/mL or 10 ng/mL canine Link-N (LN), or 0.5 μg/mL or 5 ng/mL canine short Link-N (sLN) for 28 days in hypoxia (5% O_2_). (**a**) GAG content, (**b**) DNA content, (**c**) GAG content (incorporation in the micro-aggregate) corrected for DNA content, (**d**) total amount of GAGs released in the culture medium, (**e**) representative histological images of the Safranin O/Fast Green staining and collagen type I and II immunohistochemistry. *: *p* < 0.05; xx: significantly different (*p* < 0.01) from all other conditions. *n* = 6 (*in duplicates*).

The bovine CLC micro-aggregates’ GAG content was slightly increased by 1 μg/mL canine Link-N and 0.5 μg/mL canine sLink-N treatment compared with controls (*p*<0.05; Fig 6a). The GAG content of micro-aggregates treated with 1 μg/mL canine Link-N and 0.5 μg/mL canine sLink-N was significantly higher than that of the micro-aggregates treated with 10 ng/mL canine Link-N and 5 ng/mL canine sLink-N, indicating a concentration-dependent effect (p<0.05). TGF-β_1,_ however, induced the most potent increase in GAG content (*p*<0.01). GAG incorporation percentages were not significantly different between conditions (S2D Fig). Safranin O/Fast Green staining showed the presence of GAGs in all conditions, but most prominently in the TGF-β_1_-treated micro-aggregates (Fig 6e). Treatment with canine (s)Link-N did not increase the micro-aggregates’ DNA content compared with controls, whereas TGF-β_1_ significantly increased the DNA content compared with all other conditions (*p*<0.01; Fig 6b). The GAG/DNA content was not different between conditions (Fig 6c). TGF-β_1_ induced the highest GAG release compared with all other conditions (*p*<0.01), whereas the GAG release was decreased with 10 ng/mL canine Link-N compared with controls and compared with 5 ng/mL sLink-N treatment (*p*<0.05; Fig 6d). Collagen type I was present in all micro-aggregates, but most prominent in the canine (s)Link-N-treated ones (Fig 6e). Treatment with canine (s)Link-N prominently increased collagen type II deposition compared with controls and TGF-β_1_-treated micro-aggregates (Fig 6e).

### Canine (s)Link-N induced negligible GAG deposition by CD canine CLCs

**Fig 7:**
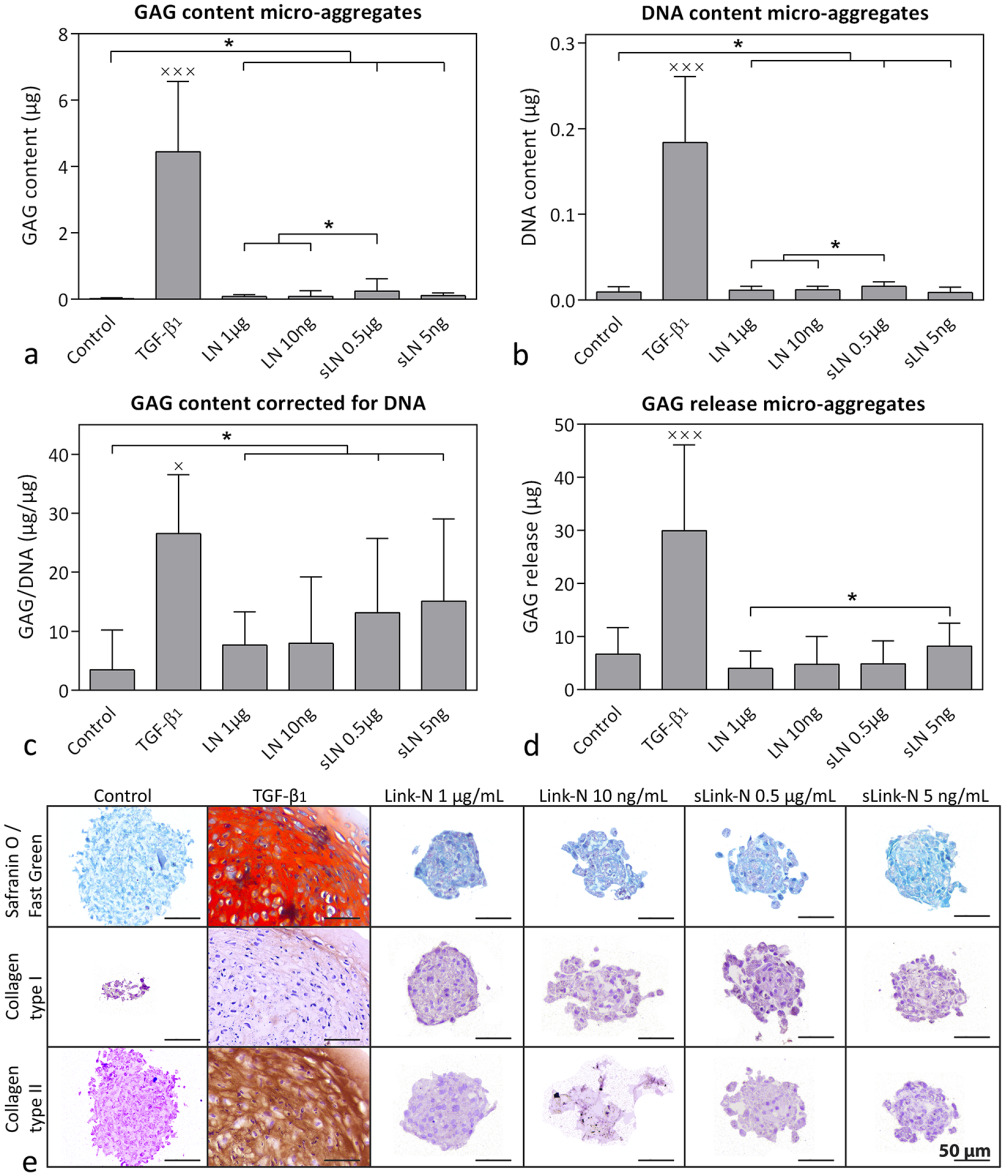
Effect of canine (short) Link-N on CD canine chondrocyte-like cells (CLCs). GAG and DNA content (mean + SD) and histological evaluation of CD canine CLC micro-aggregates treated with basal culture medium (control), supplemented with 10 ng/mL TGF-β_1_, 1 μg/mL or 10 ng/mL canine Link-N (LN), or 0.5 μg/mL or 5 ng/mL canine short Link-N (sLN) for 28 days in hypoxia (5% O_2_). (**a**) GAG content, (**b**) DNA content, (**c**) GAG content (incorporation in the micro-aggregate) corrected for DNA content, (**d**) total amount of GAGs released in the culture medium, (**e**) representative histological images of the Safranin O/Fast Green staining and collagen type I and II immunohistochemistry. *: *p* < 0.05; x,xxx: significantly different (*p* < 0.05 and *p* < 0.001 respectively) from all other conditions. *n* = 6 (*in duplicates*).

CD canine CLC micro-aggregates treated with 1 μg/mL canine Link-N and 5 ng/mL and 0.5 μg/mL canine sLink-N showed a slight, but significantly higher GAG, DNA, and GAG/DNA content compared with controls (*p*<0.05; Fig 7a–7c). TGF-β_1,_ however, induced by far the highest GAG, DNA, and GAG/DNA content (*p*<0.05; Fig 7a–7c), which was confirmed by Safranin O/Fast Green staining (Fig 7e). TGF-β_1_ also induced the highest GAG incorporation in the micro-aggregates (*p*<0.05), whereas canine (s)Link-N did not significantly increase GAG incorporation compared with controls (S2F Fig). Histological analysis indicated that canine (s)Link-N decreased the micro-aggregates size compared with controls (Fig 7e). Canine (s)Link-N did not affect GAG release compared with controls, whereas TGF-β_1_ significantly increased release compared with all other conditions (*p*<0.001; Fig 7d). Collagen type I and II deposition was not influenced by canine (s)Link-N treatment, whereas TGF-β_1_ induced a collagen type I-rich rim and prominently increased collagen type II deposition (Fig 7e).

### Human and canine (s)Link-N do not induce GAG deposition by NCD canine CLCs

Based on physical appearance, dog breeds can be divided into chondrodystrophic (CD) and non-chondrodystrophic (NCD). CD dogs have short bowlegs due to disrupted endochondral ossification. This polygenetic trait has strongly been linked with IVD degeneration. In CD dogs, replacement of NCs by CLCs in the NP starts already before one year of age and IVD disease occurs frequently. In contrast, in NCD dogs, NCs remain the predominant cell type until later in life. If IVD disease develops, it usually occurs later in life due to wear-and tear [30]. Since CD and NCD dogs differ in their genetic background and show differences in cause, prevalence, and age of onset of IVD degeneration, the regenerative potential of their CLCs could in the presence of (s)Link-N differ from each other. Therefore, we also tested the effect of (s)Link-N on NCD canine CLCs. However, both human and canine (s)Link-N did not induce any substantial effect on the GAG, DNA or GAG/DNA content or GAG release of NCD canine CLCs (S4 Fig).

### Human and canine (s)Link-N do not affect Smad signaling in human and bovine CLCs and only mildly induce Smad signaling in canine CLCs

**Fig 8:**
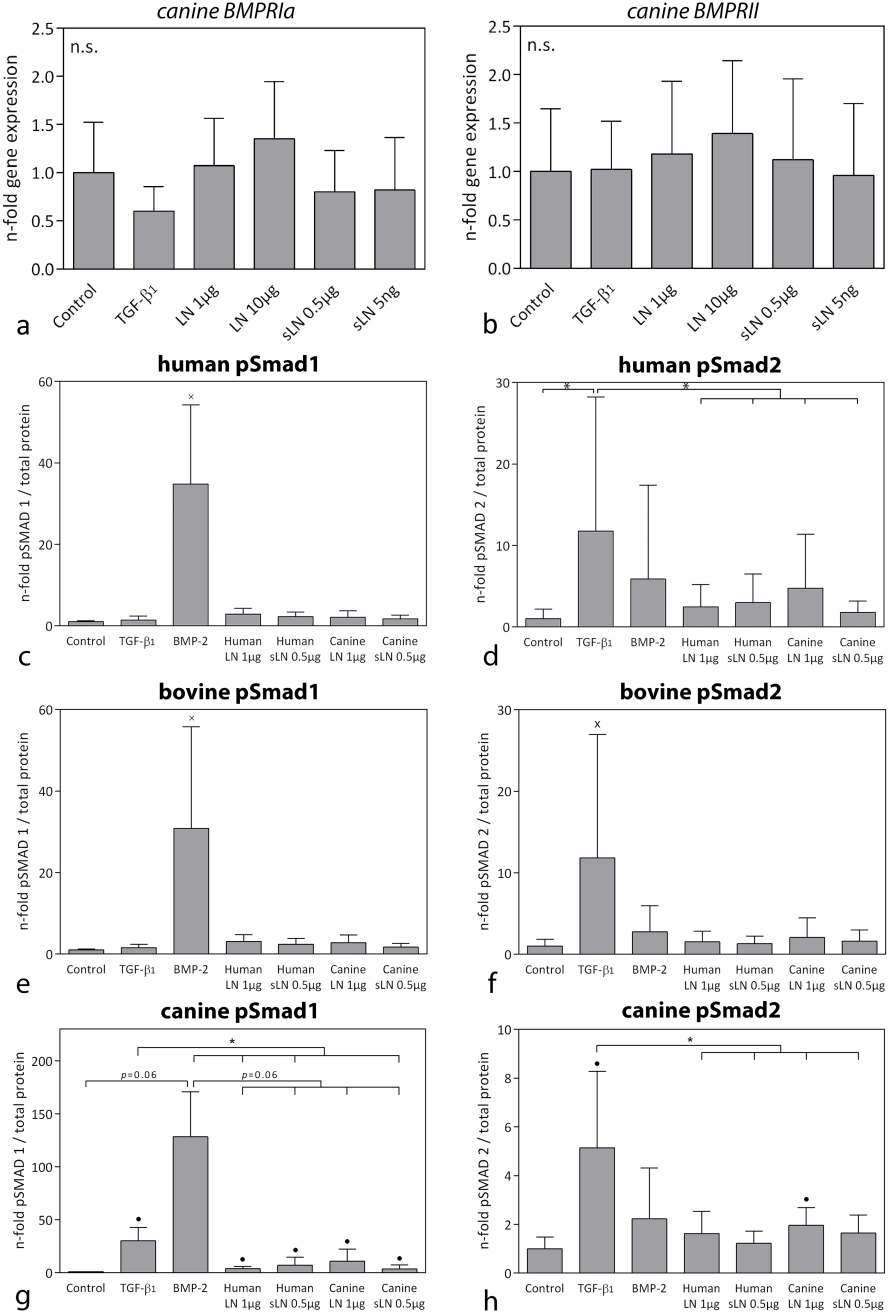
BMP receptor expression and activation of Smad signaling by (s)Link-N in human, bovine, and CD canine chondrocyte-like cells (CLCs). Relative *BMPRIa* (**a**) and *BMPRII* (**b**) gene expression (mean + SD) in CD canine CLC micro-aggregates treated with basal culture medium (control), supplemented with 10 ng/mL TGF-β_1_, 1 μg/mL or 10 ng/mL canine Link-N (LN), or 0.5 μg/mL or 5 ng/mL canine short Link-N (sLN) for 7 days in hypoxia (5% O_2_). Controls were set at 1. *n* = 6. Phosphorylated (p)Smad 1 and 2 levels in human (**c, d**), bovine (**e, f**), and canine (**g, h**) CLCs cultured in monolayers stimulated with 1 μg/mL human and canine Link-N (LN) and 0.5 μg/mL human and canine short Link-N (sLN) in hypoxia (5% O_2_) for 24 hours. *: *p* < 0.05; ●: significantly different (*p* < 0.05) from controls; x: significantly different (*p* < 0.05) from all other conditions. *n* = 5.

A possible reason for the limited response of canine CLCs to (s)Link-N could be that canine CLCs do not express BMPRII or that Smad signaling was not properly induced. Gene expression analysis, however, indicated that CD canine CLCs expressed *BMPRII* and *BMPRIa*, whereas no *BMPRIb* mRNA was detected (Fig 8a and 8b). *BMPRII* and *BMPR1a* gene expression was not significantly affected by canine (s)Link-N treatment.

Surprisingly, canine and human (s)Link-N did not significantly increase pSmad1 or pSmad2 protein levels in human and bovine CLCs after 24 hours of treatment (Fig 8c–8f). Both human and canine (s)Link-N, however, induced a mild, but significant increase in pSmad1 levels in canine CLCs (*p<*0.05; Fig 8g). Only 1 μg/mL canine Link-N mildly induced pSmad2 levels in canine CLCs (*p*<0.05; Fig 8h).

## Discussion

### Full length and short Link-N exerted comparable potent effects on CLCs

The current study confirms the results of previous work [13], since in all three tested species, sLink-N exerted comparable (regenerative) effects as full length Link-N. This confirms that the biological active part is maintained in the first eight amino acids of Link-N peptide. These results support the advantage of sLink-N compared to Link-N: the production costs of sLink-N are lower than that of Link-N and a smaller peptide is more amenable for optimization of the biological stability [13].

### Human (s)Link-N induced GAG deposition by human and bovine CLCs, but exerted limited effects on canine CLCs

Human (s)Link-N induced no effect on NCD canine CLC micro-aggregates. Additionally, it induced a significant, but very mild, concentration-independent increase in GAG content of CD canine CLC micro-aggregates. This increase was, however, considered negligible compared with the anabolic effect of TGF-β_1_. In the current study, human and bovine CLC micro-aggregates served as comparators for the 3D culture system employed. CLCs from these species have already been demonstrated to respond to Link-N in monolayers [11] and alginate beads [7, 13] with increased GAG [7] and collagen type I deposition [13], in line with the present study. Differences in culture conditions may explain our observation that human (s)Link-N did not induce collagen type II deposition in human and bovine CLCs, in contrast with previous reports [9, 11], while it increased the DNA content of bovine CLC micro-aggregates. The anabolic, concentration-dependent effect of human (s)Link-N on human and bovine CLCs suggests that the culture system allowed GAG deposition by (s)Link-N treatment, the (s)Link-N batch was active and that the limited response of canine CLCs could not be ascribed to inactive peptide.

### Interspecies differences in CLC response to canine (s)Link-N treatment

A potential reason for the mild response of canine CLCs to human (s)Link-N could be species differences in the (s)Link-N amino acid sequence. Indeed, the amino acid sequences of canine and bovine Link-N differ by five and three residues, respectively, when compared to human Link-N. Additionally, the amino acid sequences of canine and bovine sLink-N are similar and vary by only one residue from human sLink-N. Each amino acid substitution potentially affects the function of a protein [31]. Particularly the substitution of the third amino acid of human (s)Link-N (histidine) by leucine in bovine/canine (s)Link-N likely influences the 3D structure, and subsequently, receptor-docking of the peptide due to polarity differences. Therefore, we decided to also study the effects of canine (s)Link-N on human, bovine, and especially canine CLCs.

In line with previous work, in our study, human (s)Link-N exerted an anabolic effect on human CLCs [7, 11, 13], whereas canine (s)Link-N exerted no anabolic effects on human CLCs. Although suggesting a species-specific requirement, bovine CLCs were able to respond to human (s)Link in our and previous studies by increasing the production of GAG [7, 9, 13], despite the difference in amino acid sequence and polarity between human and bovine (s)Link-N. Since bovine and canine Link-N do not differ in polarity and the sLink-N sequence is similar, we hypothesized that canine (s)Link-N would exert an even more potent regenerative effect on bovine CLCs than human (s)Link-N. The present study, however, showed that canine (s)Link-N only slightly increased GAG deposition, whereas it mainly induced collagen type I and II deposition in bovine CLCs, in contrast to the response to human (s)Link-N. Thus, canine (s)Link-N may activate other pathways than human (s)Link-N in bovine CLCs. Altogether, abovementioned findings imply that species differences in amino acid sequence cannot only determine whether CLCs of a specific species respond to (s)Link-N or not, but can also determine the direction of the CLC response to (s)Link-N. To confirm or reject this hypothesis, future studies should look into the (difference in) specific pathways that are influenced by species-specific (s)Link-N in CLCs from different species.

Since human (s)Link-N exerted only a limited anabolic effect on canine CLCs, we envisioned to optimize the potency of this treatment by using canine (s)Link-N. The species-specific (s)Link-N, however, also only exerted a minor anabolic effect on CD canine CLCs and no effect on NCD canine CLCs. Thus, the results of this study indicate that both canine and human (s)Link-N do not have the potency to be used as a regenerative therapy for canines with IVD disease. Moreover, this implies that the dog cannot serve as a valid large animal model for (s)Link-N treatment of human IVD degeneration.

### Human and canine (s)Link-N slightly increased Smad signaling in canine CLCs

As human Link-N was shown to exert its effects on rabbit NCs via BMPRII by increased Smad1/5/8 signaling [12], possible reasons for the limited response of canine CLCs to (s)Link-N are insufficient expression of BMPRII and/or not properly induced Smad signaling. In the present study, canine CLCs expressed *BMPRII* mRNA, although this does not necessarily indicate that the protein is expressed at the cell surface. Nonetheless, (s)Link-N mildly induced Smad signaling in canine CLCs, indicating that the canine CLCs showed a receptor-mediated effect. In contrast to rabbit NCs and canine CLCs, human and bovine CLCs did not demonstrate increased pSmad1 or -2 levels after 24 hours of human or canine (s)Link-N treatment. This discrepancy can possibly be explained by species differences and/or different cell types present in the NP (rabbit—NCs, human/bovine—CLCs). It remains to be determined through which signaling pathways human (s)Link-N induces its effects, other than via Smad signaling in human and bovine CLCs. Notably, while human/canine (s)Link-N mildly induced Smad signaling in canine CLCs, it did not induce regenerative effects. Taken together, the results of this study indicate that (s)Link-N signaling is species-specific. Additionally, it appears that (s)Link-N can act via a yet unknown receptor besides BMPRII in human and bovine CLCs which is not or hardly present in canine CLCs. Therefore, future studies should look into the efficacy of binding of (s)Link-N to different receptors in the different species.

## Conclusions

The current study demonstrates that human and canine (s)Link-N exerted species-specific effects on CLCs from early degenerated IVDs. Although human (s)Link-N induced GAG deposition in human and bovine CLCs, canine (s)Link-N did not affect ECM production in human CLCs and mainly induced collagen deposition in bovine CLCs. Both canine and human (s)Link-N, however, did not have the potency to be used as a regenerative therapy for canines with IVD disease. This implies that the dog cannot serve as a large animal model for (s)Link-N treatment of human IVD degeneration. From a clinical perspective, the present study underscores the importance of testing the validity of animals that serve as a model for human IVD degeneration.

## Supporting information

S1 Fig Concentration range human sLink-N on CD canine CLCs. (PDF)

S2 Fig The effect of human and canine (s)Link-N on GAG incorporation. GAG incorporation ratio (mean + SD) of human, bovine, and CD canine CLC micro-aggregates treated with basal culture medium (control), supplemented with 10 ng/mL TGF-β_1_ (positive control), 1 μg/mL or 10 ng/mL human or canine Link-N (LN) or 0.5 μg/mL or 5 ng/mL human or canine sLink-N (sLN). The CLC micro-aggregates were cultured for 28 days. GAG incorporation percentages were calculated as the GAG content of the micro-aggregate divided by the total amount of GAGs produced by that micro-aggregate (GAGs released in the culture medium + GAG content of the micro-aggregate). ***: significantly different from controls. Bars indicate significant differences between conditions (*p* < 0.05). *n* = 3, (human) or 6 (bovine and canine), all in duplicates. ND: not determined, since GAG release was below the detection limit.
(PDF)

S3 Fig The effect of human (s)Link-N on canine CLCs in hypoxia vs. normoxia. GAG and DNA content (mean + SD) of CD canine CLC micro-aggregates treated with basal culture medium (control), supplemented with 10 ng/mL TGF-β_1_ (positive control), 1 μg/mL human Link-N (LN) or 0.5 μg/mL human sLink-N (sLN). The CLC micro-aggregates were cultured for 28 days in normoxia (Nx, 21% O_2_) or hypoxia (Hx, 5% O_2_). (**a**) GAG content (**b**) DNA content (**c**) GAG content corrected for DNA content (**d**) Total amount of GAGs released in the culture medium. * *p* < 0.05, ** *p* < 0.01, *** *p* < 0.001. x, xx, xxx: significantly different (*p* < 0.05, *p* < 0.01, *p* < 0.001 respectively) from all other conditions (growth factor treatment) in either Hx or Nx. *n* = 6 (in duplicates).
(PDF)

S4 Fig The effect of human and canine (s)Link-N on non-chondrodystrophic canine CLCs. Effect of human and canine (short) Link-N on non-chondrodystrophic (NCD) canine chondrocyte-like cells (CLCs). The NCD canine CLC micro-aggregates were treated with basal culture medium (control), supplemented with 10 ng/mL TGF-β1, 1 μg/mL or 10 ng/mL canine or human Link-N (LN), or 0.5 μg/mL or 5 ng/mL human or canine short Link-N (sLN) for 28 days in hypoxia (5% O_2_). (**a**) GAG content, (**b**) DNA content, (**c**) GAG content corrected for DNA content, (**d**) total amount of GAGs released in the culture medium. *, ***: significantly different from all other conditions (*p* < 0.05, *p*< 0.001, respectively). *n* = 6 (in duplicates).
(PDF)
