# Supplementary material for: Link-N: The missing link towards intervertebral disc repair is species-specific
Source: PLoS One. 2017 Nov 8;12(11):e0187831. doi: 10.1371/journal.pone.0187831 (PMC5679057; doi:10.1371/journal.pone.0187831)
Supplement: S1 Fig — (PDF) [file pone.0187831.s001.pdf]

Supplementary File 1. Concentration range human sLink-N on CD canine CLCs

Before the start of the current study, a concentration range of human (s)Link-N was tested on one CD canine CLC donor (Beagle, 2 years of age). One hundred fifty thousand CLCs were seeded in 6-well plates and treated with the indicated concentrations of sLink-N for 24 hrs. Conditioned media was collected and quantified for GAG content. 0.5-5000 ng/mL sLink-N increased GAG release in the culture medium. An optimal GAG release was established at 50-5000 ng/mL sLink-N:

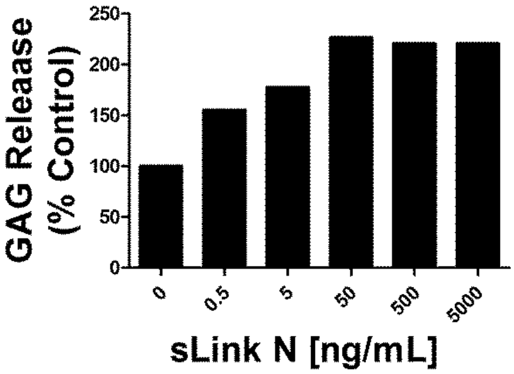

Additionally, 150.000 CLCs were seeded in 6-well plates and treated with the indicated concentrations of sLink N for 7 days. Protein was extracted in RIPA buffer and processed for Western blotting. Decreased G1 fragment indicates less aggrecan degradation.

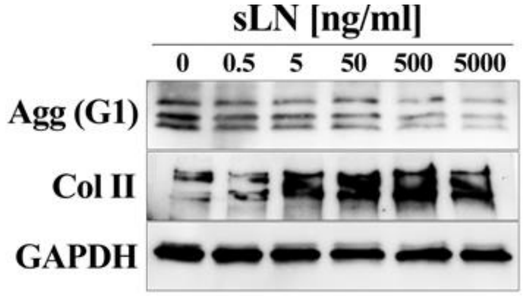

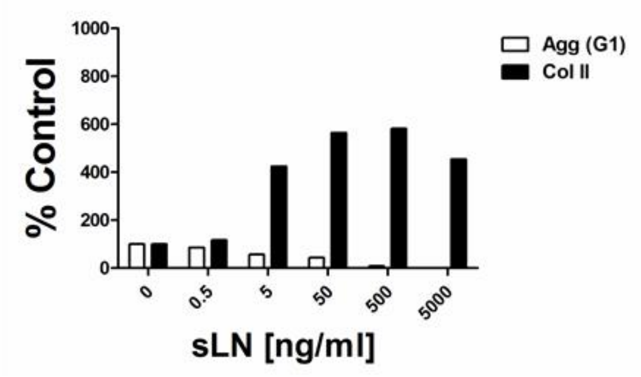

Collagen type II deposition was increased from 5 ng/mL sLink-N onwards. However, 50-5000 ng/mL did not seem to further increase collagen type II deposition. Aggrecan degradation was concentration-dependently decreased with 5 ng/mL sLink-N onwards. For these reasons, we chose to apply 5 and 500 ng/mL sLink-N and corresponding Link-N concentrations (10 and 1000 ng/mL) in the 3D micro-aggregates in the manuscript. Since culturing CLCs in a 3D environment maintains their disc phenotype better than 2D culture, 3D micro-aggregates were used to determine the effect of (s)Link-N in the current study.
