# Supplementary material for: Link-N: The missing link towards intervertebral disc repair is species-specific
Source: PLoS One. 2017 Nov 8;12(11):e0187831. doi: 10.1371/journal.pone.0187831 (PMC5679057; doi:10.1371/journal.pone.0187831)
Supplement: S2 Fig — GAG incorporation ratio (mean + SD) of human, bovine, and CD canine CLC micro-aggregates treated with basal culture medium (control), supplemented with 10 ng/mL TGF-β1 (positive control), 1 μg/mL or 10 ng/mL human or canine Link-N (LN) or 0.5 μg/mL or 5 ng/mL human or canine sLink-N (sLN). The CLC micro-aggregates were cultured for 28 days. GAG incorporation percentages were calculated as the GAG content of the micro-aggregate divided by the total amount of GAGs produced by that micro-aggregate (GAGs released in the culture medium + GAG content of the micro-aggregate). ***: significantly different from controls. Bars indicate significant differences between conditions (p < 0.05). n = 3, (human) or 6 (bovine and canine), all in duplicates. ND: not determined, since GAG release was below the detection limit. (PDF) [file pone.0187831.s002.pdf]

Supplementary File 2. The effect of human and canine (s)Link-N on GAG incorporation

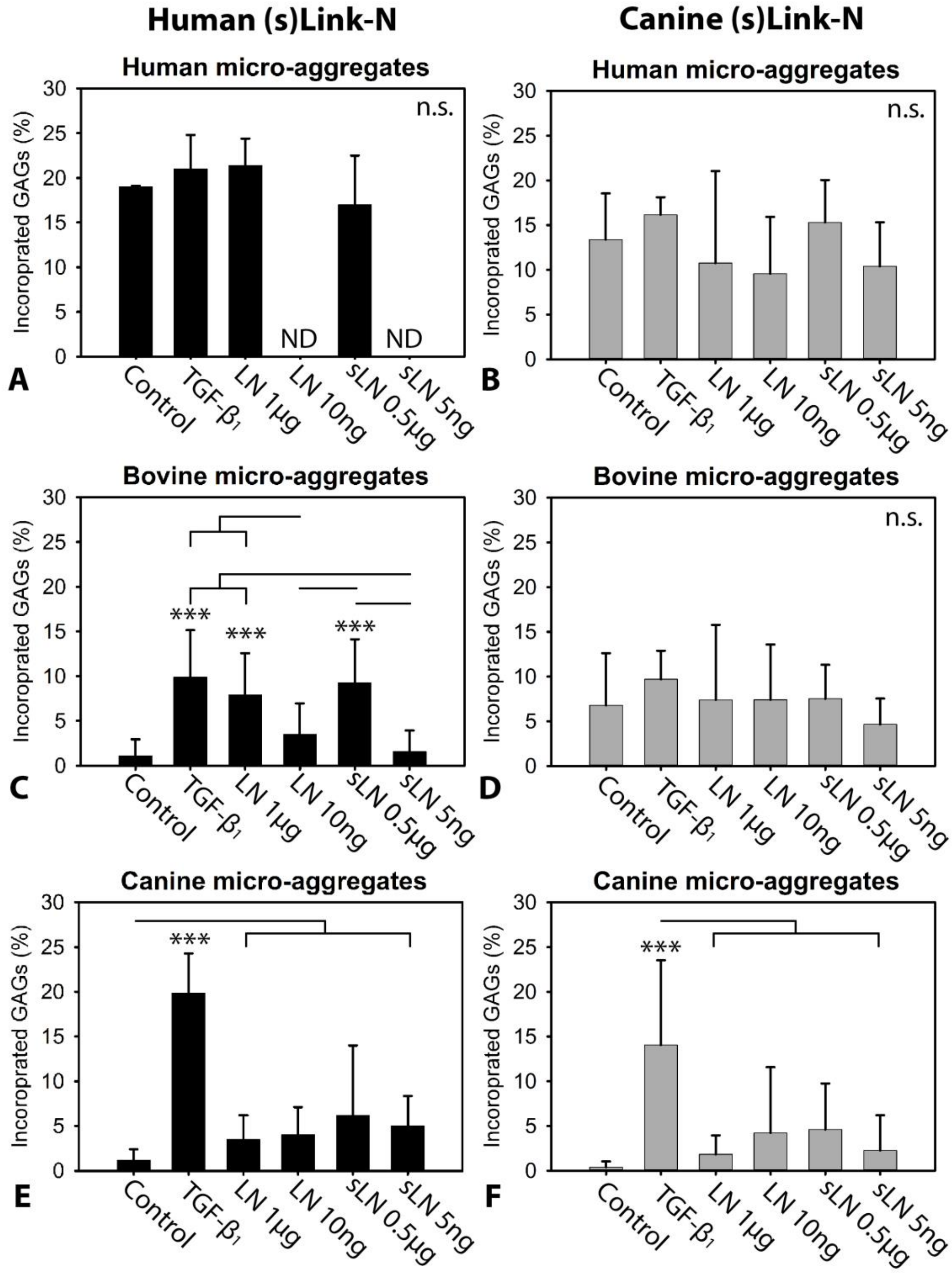

GAG incorporation ratio (mean + SD) of human, bovine, and CD canine CLC micro-aggregates treated with basal culture medium (control), supplemented with 10 ng/mL TGF-β_1 (positive control), 1 µg/mL or 10 ng/mL human or canine Link-N (LN) or 0.5 µg/mL or 5 ng/mL human or canine sLink-N (sLN). The CLC micro-aggregates were cultured for 28 days. GAG incorporation percentages were calculated as the GAG content of the micro-aggregate divided by the total amount of GAGs produced by that micro-aggregate (GAGs released in the culture medium + GAG content of the micro-aggregate). ***: significantly different from controls. Bars indicate significant differences between conditions ($p<0.05$). n = 3, (human) or 6 (bovine and canine), all in duplicates. ND: not determined, since GAG release was below the detection limit.
