# Supplementary material for: Link-N: The missing link towards intervertebral disc repair is species-specific
Source: PLoS One. 2017 Nov 8;12(11):e0187831. doi: 10.1371/journal.pone.0187831 (PMC5679057; doi:10.1371/journal.pone.0187831)
Supplement: S3 Fig — GAG and DNA content (mean + SD) of CD canine CLC micro-aggregates treated with basal culture medium (control), supplemented with 10 ng/mL TGF-β1 (positive control), 1 μg/mL human Link-N (LN) or 0.5 μg/mL human sLink-N (sLN). The CLC micro-aggregates were cultured for 28 days in normoxia (Nx, 21% O2) or hypoxia (Hx, 5% O2). (a) GAG content (b) DNA content (c) GAG content corrected for DNA content (d) Total amount of GAGs released in the culture medium. * p < 0.05, ** p < 0.01, *** p < 0.001. x, xx, xxx: significantly different (p < 0.05, p < 0.01, p < 0.001 respectively) from all other conditions (growth factor treatment) in either Hx or Nx. n = 6 (in duplicates). (PDF) [file pone.0187831.s003.pdf]

Supplementary File 3. The effect of human (s)Link-N on CD canine CLCs in hypoxia vs. normoxia

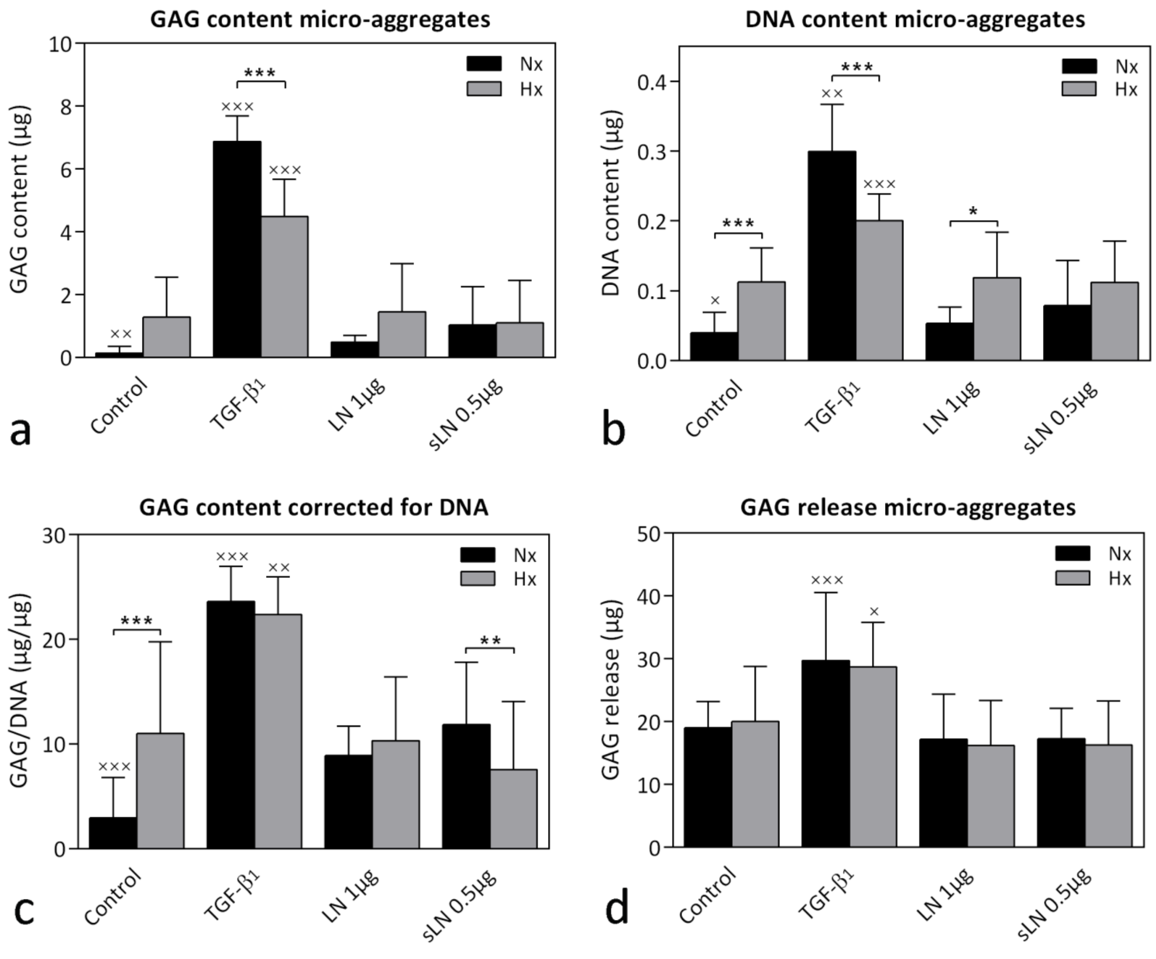

GAG and DNA content (mean + SD) of CD canine CLC micro-aggregates treated with basal culture medium (control), supplemented with 10 ng/mL TGF-β_1 (positive control), 1 µg/mL human Link-N (LN) or 0.5 µg/mL human sLink-N (sLN). The CLC micro-aggregates were cultured for 28 days in normoxia (Nx, 21% O_2) or hypoxia (Hx, 5% O_2). (**a**) GAG content (**b**) DNA content (**c**) GAG content corrected for DNA content (**d**) Total amount of GAGs released in the culture medium. ∗ $p < 0.05$, ∗∗ $p < 0.01$, ∗∗∗ $p < 0.001$. × , ×× , ××× : significantly different ($p < 0.05$, $p < 0.01$, $p < 0.001$ respectively) from all other conditions (growth factor treatment) in either Hx or Nx. $n = 6$ (in duplicates).
