# Supplementary material for: Link-N: The missing link towards intervertebral disc repair is species-specific
Source: PLoS One. 2017 Nov 8;12(11):e0187831. doi: 10.1371/journal.pone.0187831 (PMC5679057; doi:10.1371/journal.pone.0187831)
Supplement: S4 Fig — Effect of human and canine (short) Link-N on non-chondrodystrophic (NCD) canine chondrocyte-like cells (CLCs). The NCD canine CLC micro-aggregates were treated with basal culture medium (control), supplemented with 10 ng/mL TGF-β1, 1 μg/mL or 10 ng/mL canine or human Link-N (LN), or 0.5 μg/mL or 5 ng/mL human or canine short Link-N (sLN) for 28 days in hypoxia (5% O2). (a) GAG content, (b) DNA content, (c) GAG content corrected for DNA content, (d) total amount of GAGs released in the culture medium. *, ***: significantly different from all other conditions (p < 0.05, p< 0.001, respectively). n = 6 (in duplicates). (PDF) [file pone.0187831.s004.pdf]

Supplementary File 4. The effect of human and canine (s)Link-N on non-chondrodsytrophic canine CLCs

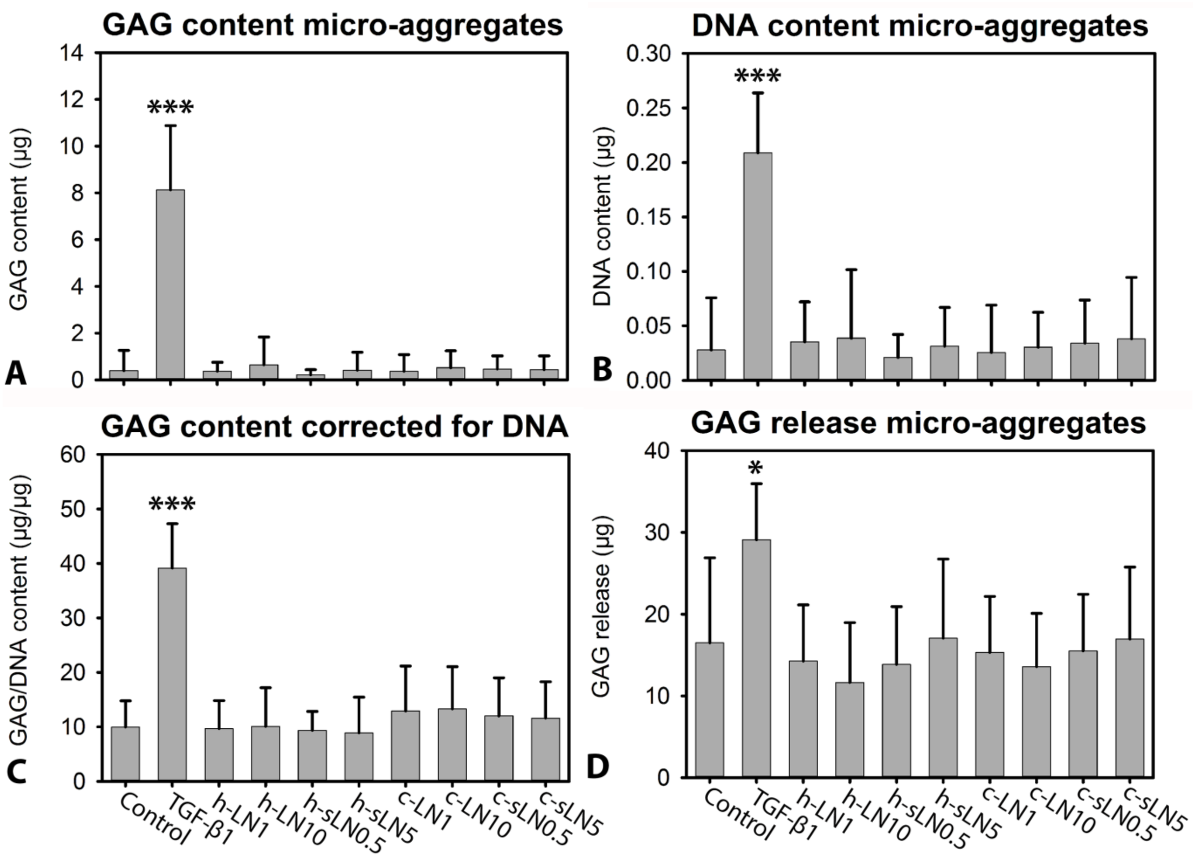

Effect of human and canine (short) Link-N on non-chondrodystrophic (NCD) canine chondrocyte-like cells (CLCs). The NCD canine CLC micro-aggregates were treated with basal culture medium (control), supplemented with 10 ng/mL TGF-β_1, 1 µg/mL or 10 ng/mL canine or human Link-N (LN), or 0.5 µg/mL or 5 ng/mL human or canine short Link-N (sLN) for 28 days in hypoxia (5% O_2). (**a**) GAG content, (**b**) DNA content, (**c**) GAG content corrected for DNA content, (**d**) total amount of GAGs released in the culture medium. *, ***: significantly different from all other conditions ($p<0.05$, $p<0.001$, respectively). $n = 6$ (in duplicates).
